# VersaVIS—An Open Versatile Multi-Camera Visual-Inertial Sensor Suite

**DOI:** 10.3390/s20051439

**Published:** 2020-03-06

**Authors:** Florian Tschopp, Michael Riner, Marius Fehr, Lukas Bernreiter, Fadri Furrer, Tonci Novkovic, Andreas Pfrunder, Cesar Cadena, Roland Siegwart, Juan Nieto

**Affiliations:** 1 Autonomous Systems Lab, ETH Zurich, 8092 Zurich, Switzerland; michael.riner-kuhn@mavt.ethz.ch (M.R.); marius.fehr@mavt.ethz.ch (M.F.); lukas.bernreiter@mavt.ethz.ch (L.B.); fadri.furrer@mavt.ethz.ch (F.F.); tonci.novkovic@mavt.ethz.ch (T.N.); cesarc@ethz.ch (C.C.); rsiegwart@ethz.ch (R.S.); nietoj@ethz.ch (J.N.); 2 Voliro Airborne Robotics, 8092 Zurich, Switzerland; 3 Sevensense Robotics, 8092 Zurich, Switzerland; andreas.pfrunder@sevensense.ch

**Keywords:** visual-inertial SLAM, time synchronization, sensor fusion, embedded, camera, IMU

## Abstract

Robust and accurate pose estimation is crucial for many applications in mobile robotics. Extending visual Simultaneous Localization and Mapping (SLAM) with other modalities such as an inertial measurement unit (IMU) can boost robustness and accuracy. However, for a tight sensor fusion, accurate time synchronization of the sensors is often crucial. Changing exposure times, internal sensor filtering, multiple clock sources and unpredictable delays from operation system scheduling and data transfer can make sensor synchronization challenging. In this paper, we present VersaVIS, an Open Versatile Multi-Camera Visual-Inertial Sensor Suite aimed to be an efficient research platform for easy deployment, integration and extension for many mobile robotic applications. VersaVIS provides a complete, open-source hardware, firmware and software bundle to perform time synchronization of multiple cameras with an IMU featuring exposure compensation, host clock translation and independent and stereo camera triggering. The sensor suite supports a wide range of cameras and IMUs to match the requirements of the application. The synchronization accuracy of the framework is evaluated on multiple experiments achieving timing accuracy of less than 1 ms. Furthermore, the applicability and versatility of the sensor suite is demonstrated in multiple applications including visual-inertial SLAM, multi-camera applications, multi-modal mapping, reconstruction and object based mapping.

## 1. Introduction

Autonomous mobile robots are well established in controlled environments such as factories where they rely on external infrastructure such as magnetic tape on the floor or beacons. However, in unstructured and changing environments, robots need to be able to plan their way and interact with their environment which, as a first step, requires accurate positioning [1]. In mobile robotic applications, visual sensors can provide solutions for odometry and Simultaneous Localization and Mapping (SLAM), achieving good accuracy and robustness. Using additional sensor modalities such as inertial measurement units (IMUs) [2,3,4] can additionally improve robustness and accuracy for a wide range of applications. For many frameworks, a time offset or delay between modalities can lead to bad results or even render the whole approach unusable. There are frameworks such as VINS-Mono [4] that can estimate a time offset during estimation, however, convergence of the estimation can be improved by accurate timestamping, that is, assigning timestamps to the sensor data that precisely correspond to measurement time from a common clock across all sensors. For a reliable and accurate sensor fusion, all sensors need to provide timestamps matchable between sensor modalities. Typically, the readout of camera image and IMU measurement are not on the same device resulting in a clock offset between the two timestamps which is hard to predict due to universal serial bus (USB) buffer delay, operating system (OS) scheduling, changing exposure times and internal sensor filtering. Therefore, measurement correspondences between modalities are ambiguous and assignment on the host is not trivial. Some of those challenges can be improved on using passive synchronization algorithms [5]. However, unobservable time delays still remain. TriggerSync [6] proposes a synchronization framework for triggered sensors that does not require additional hardware. However, this only works when all sensors are triggered simultaneously, therefore rendering exposure compensation impossible (see Section 2.1.1). Furthermore, TriggerSync is not robust against wrong association of trigger pulses due to unexpected delays and can achieve high accuracy only when a low latency connection to the host computer is available such as RS-232 [7]. A very recent combined software-hardware synchronization method is described by Lu et al. [8] which is also based on strictly simultaneously triggered sensors not taking changing exposure times into account.

Currently, there is no reference sensor synchronization framework for data collection, which makes it hard to compare results obtained in various publications that deal with visual-inertial (VI) SLAM.

Commercially and academically established sensors such as the Skybotix VI-Sensor [9] used in the EuROC micro aerial vehicle (MAV) dataset [10] and the PennCOSYVIO dataset [11], Intel RealSense [12], SkyAware sensor based on work from Honegger et al. [13] or PIRVS [14] are either unavailable and/or are limited in hardware configuration regarding image sensor, lens, camera baseline and IMU. Furthermore, it is often impossible to add further extensions to those frameworks to enable fusion with other modalities such as Light Detection and Ranging sensor (LiDAR) sensors or external illumination.

Different public datasets such as the KITTI dataset [15], the North Campus Long-Term (NCLT) dataset [16] or the Zurich Urban MAV dataset [17] all feature vision and inertial sensors. However, the sensor modalities are not synchronized in hardware rendering multiple VI SLAM approaches challenging. The most similar work to ours can be found in the Technische Universität München (TUM) Visual-Inertial dataset [18]. They combine two cameras triggered on a Genuino 101 with an IMU read out by the same microcontroller unit (MCU). Exposure times are estimated using an on-board light sensor. Unfortunately, the sensor system was not evaluated, it is not publicly available and details about exposure-time compensation, host synchronization and overall time synchronization accuracy are omitted.

**Figure 1:**
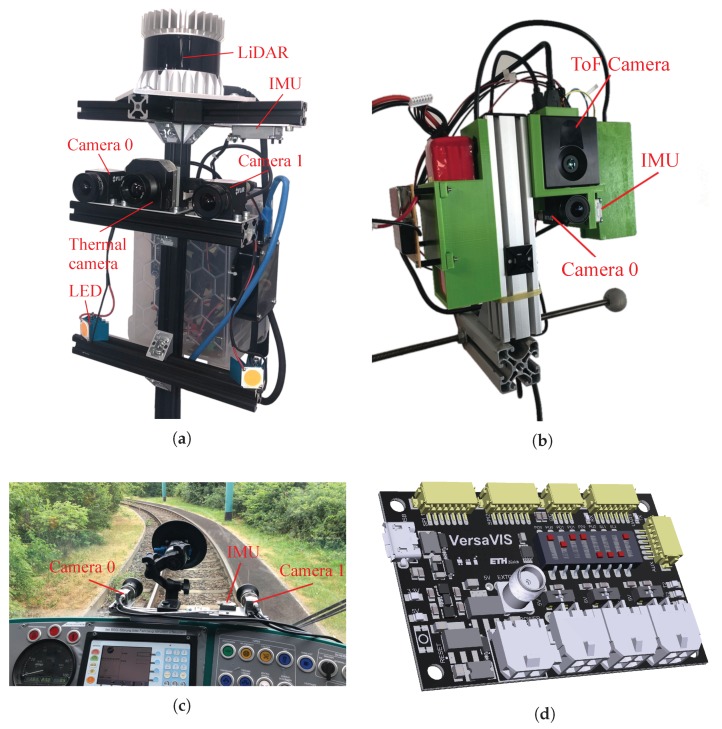
The VersaVIS sensor in different configurations. VersaVIS is able to synchronize multiple sensor modalities such as inertial measurement units (IMU) and cameras (e.g., monochrome, color, ToF and thermal) but can also be used in conjunction with additional sensors such as LiDARs. (**a**) Lidarstick; (**b**) RGB-D-I sensor; (**c**) Stereo VI Sensor [21]; (**d**) VersaVIS triggering board.

In this paper, we introduce VersaVIS (available at: www.github.com/ethz-asl/versavis) shown in Figure 1, the first open-source framework that is able to accurately synchronize a large range of camera and IMU sensors. The sensor suite is aimed for the research community to enable rapid prototyping of affordable sensor setups in different fields in mobile robotics where visual navigation is important. Special emphasis is put on an easy integration for different applications and easy extensibility by being based on well-known open-source frameworks such as Arduino [19] and the Robot Operating System (ROS) [20].

The remainder of the paper is organized as follows—in Section 2, the sensor suite is described in detail including all of its features. Section 3 provides evaluations of the synchronization accuracy of the proposed framework. Finally, Section 4 showcases the use of the Open Versatile Multi-Camera Visual-Inertial Sensor Suite (VersaVIS) in multiple applications while Section 5 provides a conclusion with an outlook on future work.

## 2. The Visual-Inertial Sensor Suite

**Figure 2:**
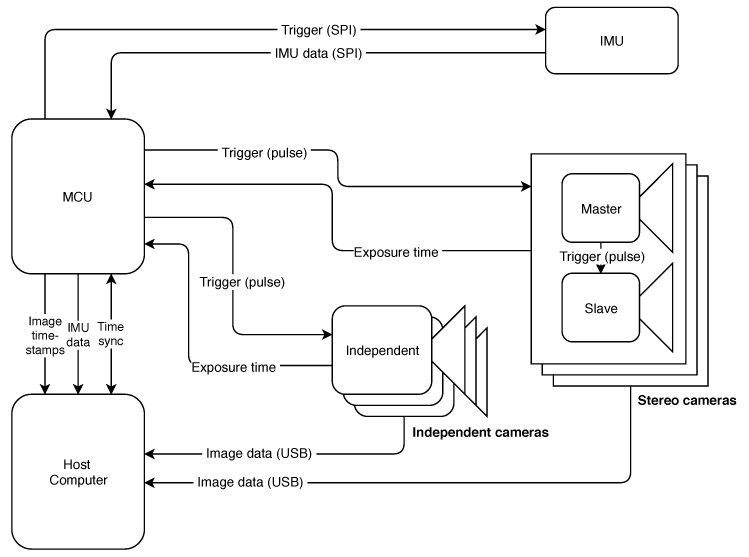
Design overview of VersaVIS. The microcontroller unit (MCU) embedded on the triggering board visible in Figure 1d sends triggers to both IMU and the connected cameras. Image data is directly transferred to the host computer where it is combined with the timestamps from the MCU.

The proposed sensor suite consists of three different parts, (i) the firmware which runs on the MCU, (ii) the host driver running on a ROS enabled machine, and (iii) the hardware trigger printed circuit board (PCB). An overview of the framework is provided in Figure 2. Here, the procedure is described for a reference setup consisting of two cameras and a Serial Peripheral Interface (SPI) enabled IMU.

The core component of VersaVIS is the MCU. First of all, it is used to periodically trigger the IMU readout together with setting the timestamps and sending the data to the host. Furthermore, the MCU sends triggering pulses to the cameras to start image exposure. This holds for both independent cameras and stereo cameras (see Section 2.1). After successful exposure, the MCU reads the exposure time by listening to the cameras’ exposure signal in order to perform exposure compensation described in Section 2.1.1 and setting mid-exposure timestamps. The timestamps are sent to the host together with a strictly increasing sequence number. The image data is hereby sent directly from the camera to the host computer to avoid massive amounts of data through the MCU. This enables to use high-resolution cameras even with a low-performance MCU.

Finally, the host computer merges image timestamps from the MCU with the corresponding image messages based on a sequence number (see Section 2.1.1).

### 2.1. Firmware

The MCU is responsible for triggering the devices at the correct time and to capture timestamps of the triggered sensor measurements. This is based on the usage of hardware timers and external signal interrupts.

#### 2.1.1. Standard Cameras

In the scope of the MCU, standard cameras are considered sensors that are triggerable with signal pulses and with non-zero data measurement time, that is, image exposure time. Furthermore, the sensors need to provide an exposure signal (often called strobe signal) which indicates the exposure state of the sensor. While the trigger pulse and the timestamp are both created on the MCU based on its internal clock, the image data is transferred via USB or Ethernet directly to the host computer. To enable correct association of the image data and timestamp on the host computer (see Section 2.2.1), both the timestamp from VersaVIS and the image data are assigned an independent sequence number $n_{VV}$ and $n_{img}$, respectively. The mapping between these sequence numbers is determined during initialization as a simultaneous start of the cameras and the trigger board cannot be guaranteed.

**Figure 3:**
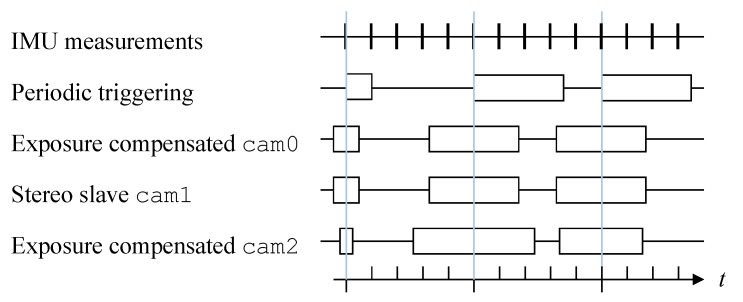
Exposure time compensation for multi-camera VI sensor setup (adapted from Reference [9]). The blue lines indicate corresponding measurements.

- Initialization procedure: After startup of the camera and trigger board, corresponding sequence numbers are found by very slowly triggering the camera without exposure time compensation. Corresponding sequence numbers are then determined by closest timestamps, see Section 2.2.1. This holds true if the triggering period time is significantly longer than the expected delay and jitter on the host computer. As soon as the sequence number offset $o_{cam}$ is determined, exposure compensation mode at full frame rate can be used.
- Exposure time compensation: Performing auto-exposure (AE), the camera adapts its exposure time to the current illumination resulting in a non-constant exposure time. Furgale et al. [22] showed, that mid-exposure timestamping is beneficial for image based state estimation, especially when using global shutter cameras. Instead of periodically triggering the camera, a scheme proposed by Nikolic et al. [9] is employed. The idea is to trigger the camera for a periodic mid-exposure timestamp by starting exposure half the exposure time earlier to its mid-exposure timestamp as shown in Figure 3 for cam0, cam1 and cam2. The exposure time return signal is used to time the current exposure time and calculate the offset to the mid-exposure timestamp of the next image. Using this approach, corresponding measurements can be obtained even if multiple cameras do not share the same exposure time (e.g., cam0 and cam2 in Figure 3).
- Master-slave mode: Using two cameras in a stereo setup compared to a monocular camera can retrieve metric scale by stereo matching. This can enable certain applications where IMU excitation is not high enough and therefore biases are not fully observable without this scale input for example, for rail vehicles described in Section 4.2. Furthermore, it can also provide more robustness. To perform accurate and efficient stereo matching, it is highly beneficial if keypoints from the same spot have a similar appearance. This can be achieved by using the exact same exposure time on both cameras. Thereby, one camera serves as the master performing AE while the other adapts its exposure time. This is achieved by routing the exposure signal from cam0 directly to the trigger of cam1 and also using it to determine the exposure time for compensation.

#### 2.1.2. Other Triggerable Sensors

Some sensors enable measurement triggering but do not require or offer the possibility to do exposure compensation for example, thermal cameras or ToF cameras. These typically do not allow for adaptive exposure compensation but rather have a fixed exposure/integration time. They can be treated the same as a standard camera, but with fixed exposure time.

Sensors that provide immediate measurements (such as external IMUs) do not need an exposure time compensation and can just use a standard timer. Note that the timestamp for those sensors are still captured on the MCU and therefore correspond to the other sensor modalities.

#### 2.1.3. Other Non-Triggerable Sensors

In robotics, it is often useful to perform sensor fusion with multiple available sensor modalities such as wheel odometers or LiDAR sensors [23]. Most of such sensor hardware do not allow triggering or low-level sensor readout but send the data continuously to a host computer. In order to enable precise and accurate sensor fusion, having corresponding timestamps of all sensor modalities is often crucial.

As most such additional sensors produce their timestamps based on the host clock or synchronized to the host clock, VersaVIS performs time *translation* to the host. In this context, clock *synchronization* refers to modifying the slave clock speed to align to the master clock while clock *translation* refers to translating the timestamp of the slave clock to the time of the master clock [7]. An on-board Kalman filter (KF) is deployed to estimate clock skew η and clock offset δ using

(1) $$t_{host}=t_{slave}+dt\cdot \eta^{k}+\delta^{k}+\Delta ,$$

where $t_{host}$ and $t_{slave}$ are the timestamps on the host and slave (in this case the VersaVIS MCU) respectively, *k* is the update step, $dt=t_{slave}-t_{slave}^{k}$ is the time since the last KF update and Δ refers to the initial clock offset set at the initial connection between host and slave.

Periodically, VersaVIS performs a filter update by requesting the current time of the host

(2) $$t_{host}^{k}=t_{host}^{a}-\frac{1}{2}\left(t_{slave}^{a}-t_{slave}^{r}\right),$$

where $t^{r}$ is the time at sending the request and $t^{a}$ is the time when receiving the answer from the host assuming that communication time delay between host and slave is symmetric. The filter update is then performed using standard KF equations:

(3) $$\begin{array}{cc} {\hat{\delta }}^{k} & =\delta^{k-1}+dt\cdot \eta^{k},\\ {\hat{\eta }}^{k} & =\eta^{k-1},\\ {\hat{P}}^{k} & =\left[\begin{array}{cc} 1 & dt\\ 0 & 1 \end{array}\right]\cdot P^{k-1}\cdot {\left[\begin{array}{cc} 1 & dt\\ 0 & 1 \end{array}\right]}^{⊤}+\left[\begin{array}{cc} Q_{\delta } & 0\\ 0 & Q_{\eta } \end{array}\right], \end{array}$$

where $\hat{\cdot }$ depicts the prediction, P is the covariance matrix and $Q_{\delta ,\eta }$ are the noise parameters of the clock offset and clock skew, respectively. The measurement residual ϵ can be written as

(4) $$\begin{array}{cc} \epsilon^{k} & =t_{host}^{k}-\Delta -t_{slave}^{k}-{\hat{\delta }}^{k}, \end{array}$$

where the Kalman gain K and the measurement update can be derived using the standard KF equations.

### 2.2. Host Computer Driver

The host computer needs to run a lightweight application in order to make sure the data from VersaVIS can be correctly used in the ROS environment.

#### 2.2.1. Synchronizer

The host computer needs to take care of merging together the image data directly from the camera sensors and the image timestamps from the VersaVIS triggering board.

During initialization, timestamps from VersaVIS $t_{VV}$ and timestamps from image data $t_{img}$ are assigned based on minimal time difference within a threshold for each connected camera separately such as

(5) $$\begin{array}{cc} n_{img},n_{VV} & =\underset{n_{img}\in N_{img},\;n_{VV}\in N_{VV}}{arg min}\left|t_{image}^{n_{VV}}-t_{VV}^{n_{VV}}\right|\\ o_{cam} & =n_{img}-n_{VV}, \end{array}$$

where $o_{cam}$ is the sequence number offset, $N_{img}$ and $N_{VV}$ are the sets of available sequence numbers from the camera and from VersaVIS, respectively, and $t^{n}$ is the timestamp corresponding to the sequence number *n*. As the images are triggered very slowly (e.g., 1 Hz), the USB buffer and OS scheduling jitter is assumed to be negligible.

As soon as $o_{cam}$ is constant and time offsets are small, the trigger board is notified about the initialization status of the camera. With all cameras initialized, normal triggering mode (e.g., high frequency) can be activated.

During normal mode, image data (directly from the camera) and image timestamps (from VersaVIS triggering board) are associated based on the sequence number like

(6) $$t_{img}^{n_{img}}\equiv t_{VV}^{n_{img}+o_{cam}}.$$

#### 2.2.2. IMU Receiver

In addition to the camera data, in a setup where the IMU is triggered and read out by the VersaVIS triggering board, the IMU message should only hold minimal information to minimize bandwidth requirements and therefore needs to be reassembled into a full IMU message on the host computer.

### 2.3. VersaVIS Triggering Board

One main part of VersaVIS is the triggering board which is a MCU-based custom PCB shown in Figure 1d that is used to connect all sensors and performs sensor synchronization. For easy extensibility and integration, the board is compatible with the Arduino environment [19]. In the reference design, the board supports up to three independently triggered cameras with a four pin connector. Furthermore, SPI, Inter-Integrated Circuit (I${}^{2}$C) or Universal Asynchronous Receiver Transmitter (UART) can be used to interface with an IMU or other sensors. Table 1 shows the specifications of the triggering board. The board is connected to the host computer using USB and communicates with ROS using *rosserial* [24].

## 3. Evaluations

In this section, several evaluations are carried out that show the synchronization accuracy of different modules of the VersaVIS framework.

### 3.1. Camera-Camera

The first important characteristic of a good multi-camera time synchronization is that multiple corresponding camera images capture the same information. This is especially important when multiple cameras are used for state estimation (see Section 4.2).

**Figure 4:**
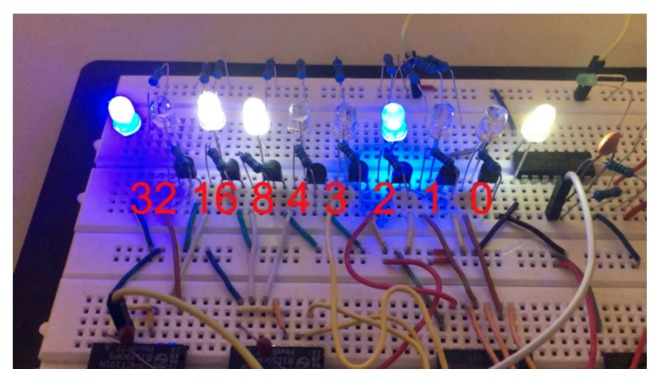
LED timing board with indicated state numbers. The left and right most LEDs are used for position reference. The four right counting LEDs are striding while the four counting LEDs on the left side are binary encoded.

For the purpose of evaluating the synchronization accuracy of multi-camera triggering, we captured a stream of images of a light emitting diode (LED) timing board shown in Figure 4 with two independently triggered but synchronized and exposure-compensated cameras. The board features eight counting LEDs (the left and right most LEDs are always on and used for position reference). The board is changing state whenever a trigger is received. The LEDs are organized in two groups. The right group of four indicates one count each, while the left group is binary encoded resulting in a counter overflow at 64. The board is triggered with $f_{l}=1\;\mathrm{kHz}$ aligned with the mid-exposure timestamps of the images. Furthermore, both cameras are operated at a rate of $f_{c}=10\;\mathrm{Hz}$ and with a fixed exposure time of $t_{c}=1\;\mathrm{ms}$.

**Figure 5:**
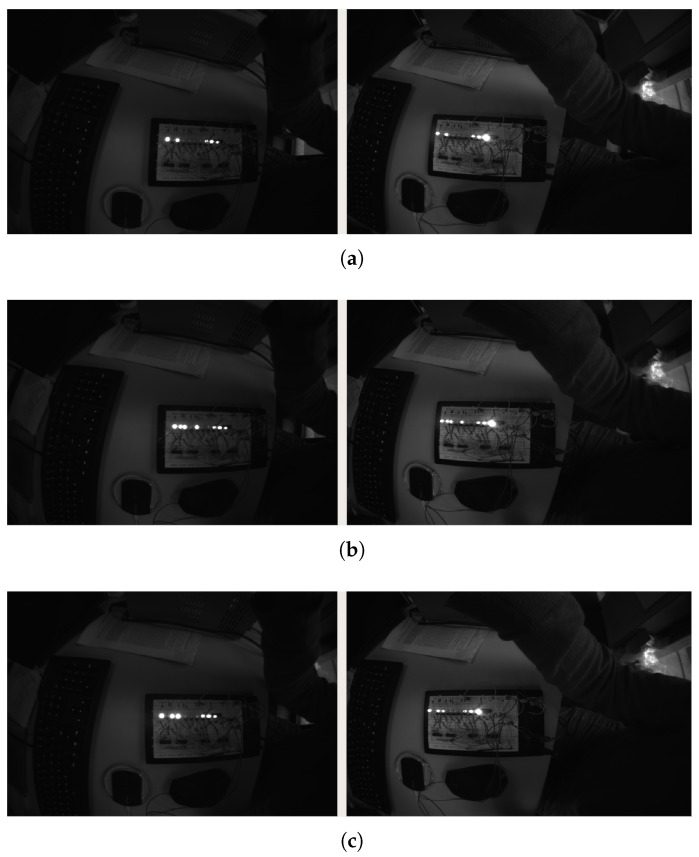
Measurements of two independently triggered synchronized cameras with exposure compensation (left cam0, right cam2). Both cameras show strictly the same image with at most two of the striding LEDs on. The increment between consecutive measurements adds up correctly and overflows at a count of 64. (**a**) LED count $c_{1}=16$; (**b**) LED count $c_{2}=c_{1}+\;$κ =116%64=52; (**c**) LED count $c_{3}=c_{2}+\;$κ =216%64=24.

For a successful synchronization of the sensors, images captured at the same time-step *i* with both cameras should show the same bit count $c_{i}$ with at most two of the striding LEDs on (since the board changes state at mid-exposure) and also the correct increment between images of κ $=\frac{f_{l}}{f_{c}}=100$. Figure 5 shows results of three consecutive image pairs. All image pairs (left and right) show the same LED count while consecutive images show the correct increment of 100. An image stream containing 400 image pairs was inspected without any case of wrong increment or non-matching pairs.

We can therefore conclude that the time synchronization of two cameras has an accuracy better than $\frac{1}{2\cdot f_{l}}=0.5\;\mathrm{ms}$ confirming an accurate time synchronization.

### 3.2. Camera-IMU

For many visual-inertial odometry (VIO) algorithms such as ROVIO [2] or OKVIS [3], accurate time synchronization of camera image and IMU measurement is crucial for a robust and accurate operation.

Typically, time offsets between camera and IMU can be the result of data transfer delay, OS scheduling, clock offsets of measurement devices, changing exposure times or internal filtering of IMU measurements. Thereby, only offsets that are not constant are critical as constant offsets can be calibrated. Namely, offsets that are typically changing such as OS scheduling, clocks on different measurement devices or a not compensated changing exposure time should be avoided.

Using the camera-IMU calibration framework Kalibr [22], a time offset between camera and IMU measurement can be determined by optimizing the extrinsic calibration together with a constant offset between both modalities. To test the consistency of the time offset, multiple datasets *N* were recorded with VersaVIS using different configurations for the IMU filtering and exposure times. The window width *B* of the deployed Barlett window finite impulse response (FIR) filter on the IMU [25] was set to B={0,2,4} while the exposure time $t_{e}$ was set to AE or fixed to $t_{e}=\left\{1,3,5\right\}\;\mathrm{ms}$.

Furthermore, also the Skybotix VI-Sensor [9] and Intel RealSense T265 [12] were tested for reference.

**Figure 6:**
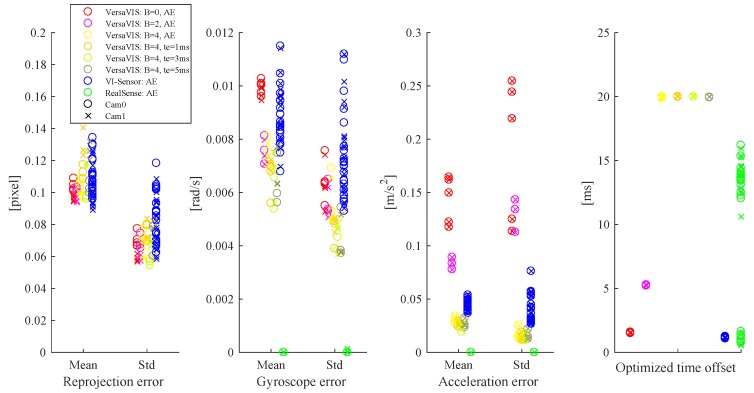
Distribution of camera to IMU calibration results using the different sensors and different sensor configurations for VersaVIS. Reprojection errors for RealSense are not visible as they are out of view, see Table 2. While reprojection, gyroscope and acceleration errors can be evaluated per measurement in each dataset and therefore shown as mean and standard deviation, the optimized time offset is one value over the whole dataset and shown as individual results.

Table 2 and Figure 6 show import indicators of the calibration quality for multiple datasets. The reprojection error represents how well the lens and distortion model fit the actual lens and how well the movement of the camera agrees with the movement of the IMU after calibration. The reprojection error of VersaVIS and VI-Sensor are both low and consistent meaning that the the calibration converged to a consistent extrinsic transformation between camera and IMU and both sensor measurements agree well. Furthermore, the reprojection errors are independent of the filter and exposure time configuration showing that exposure compensation is working as expected. On the other side, the RealSense shows high reprojection errors because of the sensor’s fisheye lenses which turn out to be hard to calibrate even with the available fisheye lens models [26] in Kalibr.

This also becomes visible in the acceleration and gyroscope errors where the errors are very low as a result to the poorly fitting lens model. Due to the high influence of the lense model on the overall objective function of camera to IMU calibration, the reprojection error part of the objective function is mainly dominated by the poorly fitting lense model. This causes erroneous gradients, and can result in a higher weighting of the transformation between body and IMU compared to the weighting of the transformation between body and camera, which is a sub-optimal local minimum. Kalibr therefore estimates the body spline to be mainly represented by the IMU and neglects image measurements. For VersaVIS, both errors are highly dependent on the IMU filter showing a decrease of error with more aggressive filtering due to minimized noise. However, also here similar or lower errors can be achieved using VersaVIS compared to the VI-Sensor.

Finally, the time offset between camera and IMU measurements shows that both VersaVIS and the VI-Sensor possess a similar accuracy in time synchronization as the standard deviations are low and the time offsets consistent indicating synchronization accuracy below 0.05 ms. However, the time offset is highly dependent on the IMU filter configuration. Therefore, the delay should be compensated either on the driver side or on the estimator side when more aggressive filtering is used (e.g., to reduce the influence of vibrations). Furthermore, the time offset is independent of exposure time and camera indicating again that exposure compensation is working as intended. RealSense shows inconsistent time offset estimations with a bi-modal distribution delimited by half the inter-frame time of ≈15 ms indicating that for some datasets, there might be image measurements shifts by one frame.

### 3.3. VersaVIS-Host

As mentioned in Section 2.1.3, not all sensors are directly compatible with VersaVIS. The better the clock translation of different measurement devices, the better the sensor fusion.

Thanks to the bi-directional connection between VersaVIS and host computer, clock translation requests can be sent from VersaVIS and the response from the host can be analyzed.

**Figure 7:**
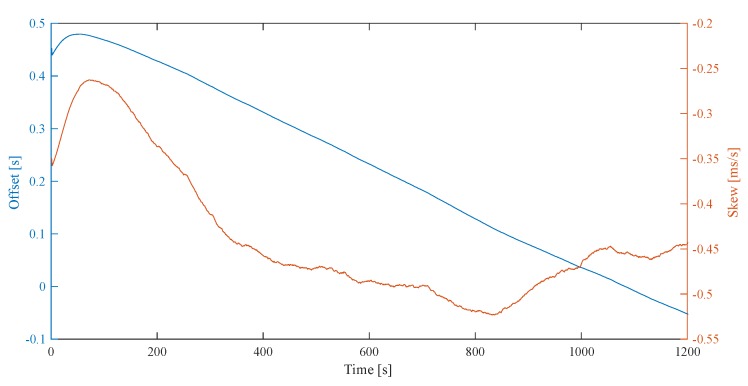
KF filter states after startup and in convergence. The offset is constantly decreasing after convergence due to a clock skew difference between VersaVIS and host computer.

Such requests are sent every second. Figure 7 shows the evolution of the KF states introduced in Section 2.1.3. In this experiment, there is a clock skew between the host computer and VersaVIS resulting in a constantly decreasing offset after KF convergence. This highlights the importance of estimating the skew when using time translation.

**Figure 8:**
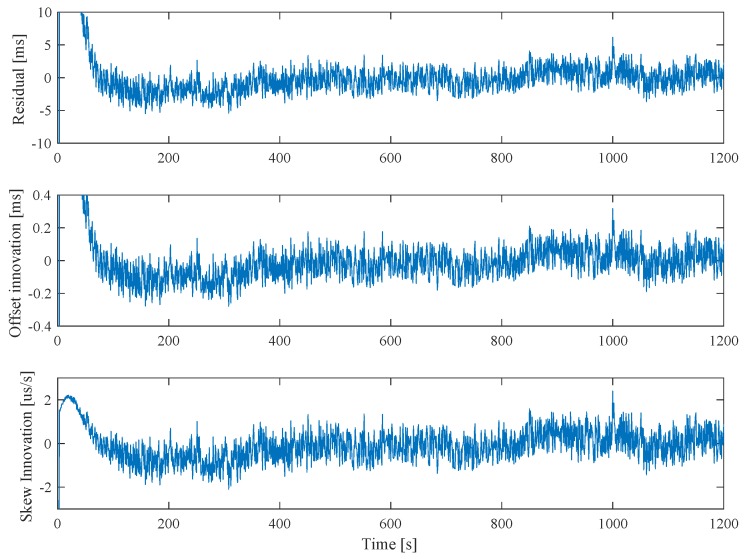
KF residual and innovation terms after startup and in convergence. The jitter in the serial-USB interface is directly influencing the residual resulting in oscillating errors. However, as this jitter has zero-mean, the achieved clock synchronization/translation has a much higher accuracy visible in the innovation term of the clock offset.

Figure 8 shows results of the residual ϵ and the innovation terms of the clock offset δ and skew η after startup and in convergence. After approximately 60 s, the residual drops below 5 ms and keeps oscillating at ±5 ms due to USB jitter. However, thanks to the KF, this error is smoothed to a zero mean innovation of the offset of ±0.2 ms resulting in a clock translation accuracy of ±0.2 ms. The influence of the skew innovation can be neglected with the short update time of 1 s.

## 4. Applications

This section validates the flexibility, robustness and accuracy of our system with several different sensor setups using VersaVIS, utilized in different applications.

### 4.1. Visual-Inertial SLAM

The main purpose of a VI sensor is to perform odometry estimation and mapping. For that purpose, we collected a dataset walking around in our lab with different sensor setups including VersaVIS equipped with a FLIR BFS-U3-04S2M-CS camera and the Analog Devices ADIS16448 IMU shown in Figure 1a, a Skybotix VI-Sensor [9] and an Intel RealSense T265 all attached to the same rigid body. For reference, we also evaluated the use of the FLIR camera together with the IMU of the VI-Sensor as a non-synchronized sensor setup. Since both sensors communicate with the host, software time translation is available.

**Figure 9:**
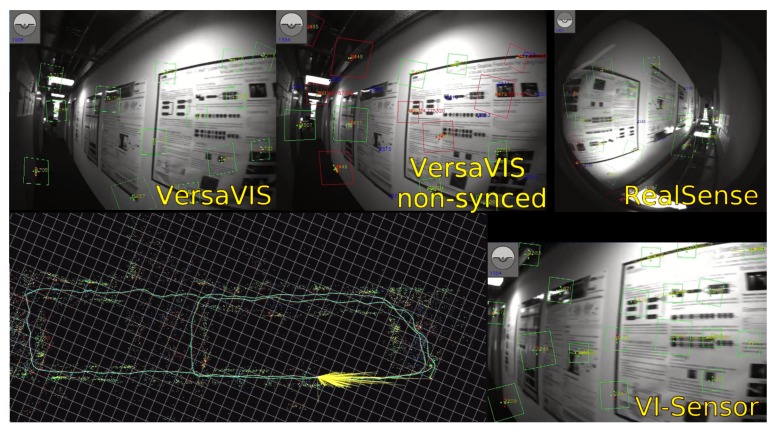
Feature tracking of ROVIO [2] on a dataset recorded in our lab. Inliers of the feature tracking are shown in green while outliers are shown in red.

Figure 9 shows an example of the feature tracking window of ROVIO [2], a filtering based monocular visual-inertial odometry algorithm, on the dataset. Both VersaVIS and the VI-Sensor show many well tracked features even during fast motions depicted on the image. Due to the high field of view (FOV) of the RealSense cameras and not perfectly fitting lens model, some of the keypoints do not reproject correctly to the image plane resulting in falsely warped keypoints. With a non-synchronized sensor (VersaVIS non-synced), many of the keypoints cannot be correctly tracked as the IMU measurements and the image measurements do not agree well.

**Figure 10:**
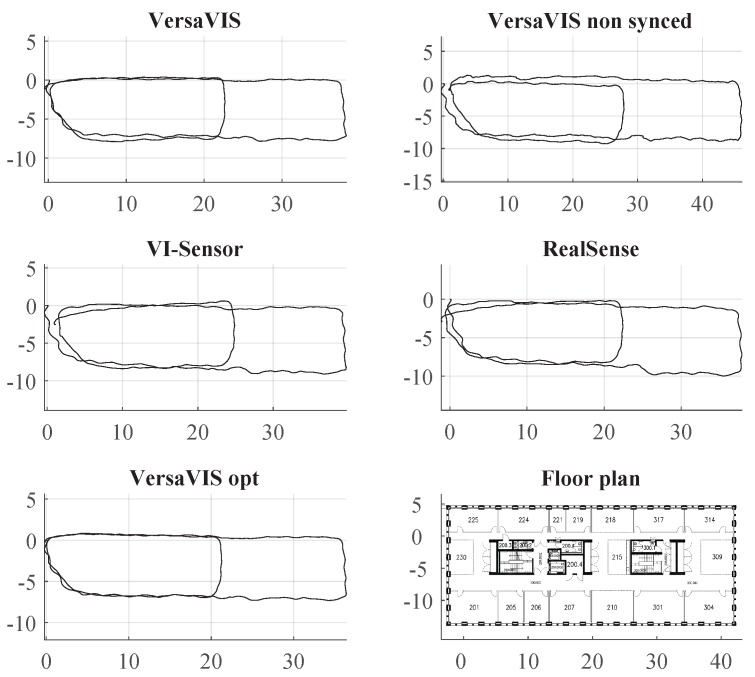
Trajectories of different sensor setups using ROVIO [2] on a dataset recorded in our lab. While all of the sensors provide a useful output, VersaVIS shows the lowest amount of drift while the non-synchronized sensor shows a lot of jitter in the estimation.

Figure 10 shows trajectories obtained with the procedure described above. While all sensors provide useful output depending on the application, VersaVIS shows the lowest drift. VI-Sensor and RealSense suffer from their specific camera hardware where the VI-Sensor has inferior lenses and camera chips (visible in motion blur) and RealSense has camera lenses where no well-fitting lens model is available in the used frameworks. Without synchronization, the trajectory becomes more jittery resulting in potentially unstable estimator also visible in the large scale offset and shows higher drift. Using batch optimization and loop closure [27], the trajectory of VersaVIS can be further optimized (VersaVIS opt). However, the discrepancy between VersaVIS and VersaVIS opt is small indicating an already good odometry performance.

### 4.2. Stereo Visual-Inertial Odometry on Rail Vehicle

The need for public transportation is heavily increasing while current infrastructure is reaching its limits. Using on-board sensors with reliable and accurate positioning, the efficiency of the infrastructure could be highly increased [21]. However, this requires the fusion of multiple independent positioning modalities of which one could be visual-aided odometry.

For this purpose, VersaVIS was combined with two global-shutter cameras arranged in a fronto-parallel stereo setup using master-slave triggering (see Section 2.1) and a compact, precision six degrees of freedom IMU shown in Figure 1c. In comparison to many commercial sensors, the camera has to provide a high frame-rate to be able to get a reasonable number of frames per displacement, even at higher speeds and feature a high dynamic range to deal with the challenging lighting conditions. Furthermore, due to the constraint motion of the vehicle and low signal to noise ratio (SNR), the IMU should be of a high quality and also should be temperature calibrated to deal with temperature changes due to direct sunlight. The sensor specifications are summarized in Table 3.

Multiple datasets were recorded with VersaVIS on a tram and evaluated against real time kinematics (RTK) global navigation satellite system (GNSS) ground-truth. The evaluation was performed using a sequence-based approach [28] and shows that by using stereo cameras and tightly synchronized IMU measurements, robustness can be improved and accurate odometry up to 1.11 % of the travelled distance evaluated on 50 m sequences on railway scenarios and speeds up to 52.4 km/h can be achieved. This corresponds to a median error of 55.5 cm per 50 m travelled. For more details, please refer to our previous work [21].

### 4.3. Multi-Modal Mapping and Reconstruction

VI mapping as described in Section 4.1 can provide reliable pose estimates in many different environments. However, for applications that require precise mapping of structures in GPS denied, visually degraded environments, such as mines and caves, additional sensors are required. The multi-modal sensor setup as seen in Figure 1a was specifically developed for mapping research in these challenging conditions. The fact that VersaVIS provides time synchronization to the host computer greatly facilitates the addition of other sensors. The prerequisite is that these additional sensors are time synchronized with the host computer as well, which in our case, an Ouster OS-1 64-beam LiDAR, is done over Precision Time Protocol (PTP). The absence of light in these underground environments also required the addition of an artificial lighting source that fulfills very specific requirements to support the camera system for pose estimation and mapping. The main challenge was to achieve the maximum amount of light, equally distributed across the environment (i.e., ambient light), while at the same time being bound by power and cooling limitations. To that end a pair of high-powered, camera-shutter-synchronized LEDs, similar to to the system shown by Nikolic et al. [29], are employed. VersaVIS provides a trigger signal to the LED control board, which represents the union of all camera exposure signals, ensuring that all images are fully illuminated while at the same time minimizing the power consumption and heat generation. This allows operating the LEDs at a significantly higher brightness level than during continuous operation.

**Figure 11:**
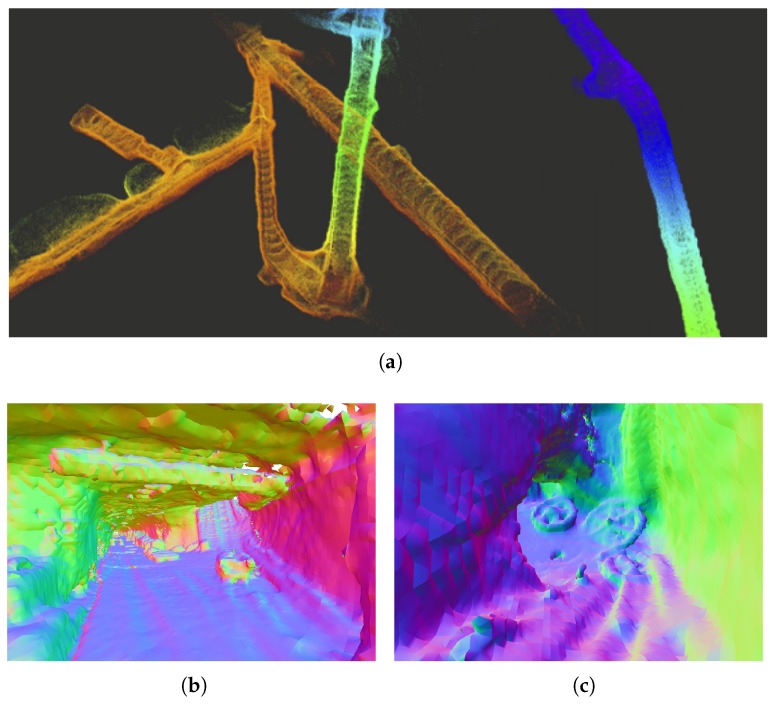
3D reconstruction of an underground mine in Gonzen CH based on LiDAR data with poses provided by VI SLAM [27]. (**a**) Accumulated points clouds using only VI SLAM poses; (**b**) Dense reconstruction example based on [30]; (**c**) Visible details of wheels lying on the ground using den se reconstruction based on Reference [30].

Figure 11 shows an example of the processed multi-modal sensor data. VI odometry [2] and mapping [27] including local refinements based on LiDAR data and Truncated Signed Distance Function (TSDF)-based surface reconstruction [30] were used to precisely map the 3D structure of parts of an abandoned iron mine in Switzerland.

### 4.4. Object Based Mapping

Robots that operate in changing environments benefit from using maps that are based on physical objects instead of abstract points. Such object based maps are more consistent if individual objects move, as only a movement of an object must be detected instead of each point on the moved object that is part of the map. Object based maps are also a better representation for manipulation tasks. The elements in the map are typically directly the objects of interest in such tasks.

For the object based mapping application, a sensor setup with a depth and an RGB camera, and an IMU was assembled, see Figure 1b. A Pico Monstar, which is a ToF camera that provides 352×287 resolution depth images at up to 60 Hz, was combined with a FLIR BFS-U3-16S2C-CS 1.6 MP color camera, and an Analog Devices ADIS16448 IMU. To obtain accurate pose estimates, the IMU and color camera were used for odometry and localization [27].

**Figure 12:**
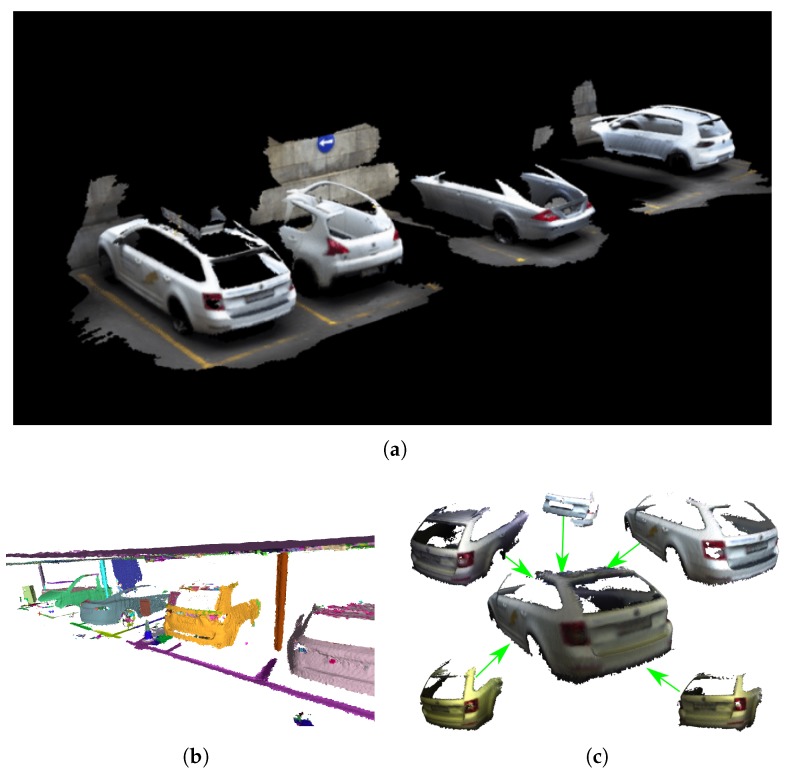
Object segmentation and reconstruction based on References [31,32] using data from the RGB-D-I sensor and camera poses obtained with maplab [27]. (**a**) Scene reconstruction using the RGB-D-I sensor; (**b**) Segmented objects in a parking garage using [31]; (**c**) Merging of objects from the database [31].

With these poses, and together with the depth measurements, an approach from Reference [31] was used to reconstruct the scene and extract object instances. An example of such a segmentation map is shown in Figure 12a, and objects that were extracted and inserted into a database in Figure 12b,c.

## 5. Conclusions

We presented a hardware synchronization suite for multi-camera VI sensors consisting of the full hardware design, firmware and host driver software. The sensor suite supports multiple beneficial features such as exposure time compensation and host time translation and can be used in both independent and master-slave multi-camera mode.

The time synchronization performance is analyzed separately for camera-camera synchronization, camera-IMU synchronization and VersaVIS-host clock translation. All modules achieve time synchronization accuracy of <1 ms which is expected to be accurate enough for most mobile robotic applications.

The benefits and great versatility range of the sensor suite are demonstrated on multiple applications including hand-held VIO, multi-camera VI applications on rail vehicles as well as large scale environment reconstruction and object based mapping.

For the benefit of the community, all hardware and software components are completely open-source with a permissive license and based on easily available hardware and development software. This paper and the accompanying framework can also serve as a freely available reference design for research and industry as it summarizes solution approaches to multiple challenges of developing a synchronized multi-modal sensor setup.

The research community can easily adopt, adapt and extend this sensor setup and rapid-prototype custom sensor setups for a variety of robotic applications. Many experimental features showcase the easy extensibility of the framework:

- Illumination module: The VersaVIS triggering board can be paired with LEDs shown in Figure 1a which are triggered corresponding to the longest exposure time of the connected cameras. Thanks to the synchronization, the LEDs can be operated at a higher brightness as which would be possible in continuous operation.
- IMU output: The SPI output of the board enables to use the same IMU which is used in the VI setup for a low-level controller such as the PixHawk [33] used in MAV control.
- Pulse per second (PPS) sync: Some sensors such as specific LiDARs allow synchronization to a PPS signal provided by for example, a Global Position System (GPS) receiver or real-time clock. Using the external clock input on the triggering board, VersaVIS can be extended to synchronize to the PPS source.
- LiDAR synchronization: The available auxiliary interface on VersaVIS could be used to tightly integrate LiDAR measurements by getting digital pulses from the LiDAR corresponding to taken measurements. The merging procedure would then be similar to the one described in Section 2.2.1 for cameras with fixed exposure time.

## Figures and Tables

**Table 1:** Hardware characteristics for the VersaVIS triggering board.

MCU	Hardware Interface	Host Interface	Weight	Size	Price
ARM M0+	SPI, I${}^{2}$C, UART	Serial-USB 2.0	15.2 g	62×40×13.4 mm	<100$

**Table 2:** Mean values of camera to IMU calibration results for different sensors and sensor configurations for VersaVIS.

	*B*	$t_{e}$	N	Reprojection Error [pixel]		Gyroscope Error [rad/s]	
				Mean	Std	Mean	Std
VersaVIS	0	AE	10	0.100078	0.064657	0.009890	0.006353
VersaVIS	2	AE	6	0.098548	0.063781	0.007532	0.005617
VersaVIS	4	AE	6	0.101866	0.067196	0.007509	0.005676
VersaVIS	4	1 ms	6	0.121552	0.075939	0.006756	0.004681
VersaVIS	4	3 ms	6	0.108760	0.062456	0.006483	0.004360
VersaVIS	4	5 ms	6	0.114614	0.074536	0.006578	0.00428
VI-Sensor	×	AE	40	0.106839	0.083605	0.008915	0.007425
Realsense	×	AE	40	0.436630	0.355895	0.000000	0.000005
	B	$t_{e}$	**N**	**Accelerometer error** $\left[m/s^{2}\right]$		**Time offset** [ms]	
				**Mean**	**Std**	**Mean**	**Std**
VersaVIS	0	AE	10	0.143162	0.191270	1.552360	0.034126
VersaVIS	2	AE	6	0.083576	0.130018	5.260927	0.035812
VersaVIS	4	AE	6	0.030261	0.018168	19.951467	0.049712
VersaVIS	4	1 ms	6	0.026765	0.014890	20.007137	0.047525
VersaVIS	4	3 ms	6	0.024309	0.014367	20.002966	0.035952
VersaVIS	4	5 ms	6	0.027468	0.016553	19.962924	0.029872
VI-Sensor	×	AE	40	0.044845	0.042446	1.173100	0.046410
Realsense	×	AE	40	0.000000	0.000002	9.884808	5.977421

**Table 3:** Sensor specifications deployed for data collection on rail vehicles [21].

Device	Type	Specification
Camera	Basler acA1920-155uc	Frame-rate 20 fps (The hardware is able to capture up to 155 fps.), Resolution 1920×1200, Dynamic range 73 dB
Lense	Edmund Optics	Focal length 8 mm≈70deg opening angle; Aperture f/5.6
IMU	ADIS16445	Temperature calibrated, 300 Hz, ±250 deg/s, $\pm 49\;m/s^{2}$
